# Impact of pre-existing disorder on radiation defect dynamics in Si

**DOI:** 10.1038/s41598-019-48415-7

**Published:** 2019-08-26

**Authors:** J. B. Wallace, L. B. Bayu Aji, L. Shao, S. O. Kucheyev

**Affiliations:** 1 Lawrence Livermore National Laboratory, Livermore, California 94550 USA; 2 Department of Nuclear Engineering, Texas A&M University, College Station, Texas 77843 USA

**Keywords:** Surfaces, interfaces and thin films, Electronic devices

## Abstract

The effect of pre-existing lattice defects on radiation defect dynamics in solids remains unexplored. Here, we use a pulsed beam method to measure the time constant of defect relaxation for 500 keV Ar ion bombardment of Si at 100 °C with the following two representative types of pre- existing lattice disorder: (i) point defect clusters and (ii) so-called “clamshell” defects consisting of a high density of dislocations. Results show that point defect clusters slow down defect relaxation processes, while regions with dislocations exhibit faster defect interaction dynamics. These experimental observations demonstrate that the dynamic aspects of damage buildup, attributed to defect trapping-detrapping processes, can be controlled by defect engineering.

## Introduction

When an energetic ion impinges on a solid, a cascade of ballistically generated point defects is created. In most practical cases, these point defects are not “frozen” in the crystal lattice but undergo so-called dynamic annealing (DA) through migration, recombination, and clustering^1,2^. Such DA processes are sensitively affected by the material microstructure. Indeed, surfaces, grain boundaries, extended defects (such as dislocations and stacking faults), and point defects can strongly influence damage buildup and material’s ability to efficiently recover from radiation damage. Perhaps the best known example of the influence of pre-existing defects on radiation damage formation is the non-linearity of damage buildup (i.e., the accumulation of defects with increasing dose) observed for most materials^1,2^.

In some cases, pre-existing defects can lead to more efficient damage recovery during irradiation and, hence, improved radiation tolerance. For example, nanocrystalline metals, including Pd^3^, Au^4^, Ni^5^, Cu-Al_2_O_3_^5^, and TiNi^6^, exhibit an increase in radiation tolerance with grain refinement. In addition, cold working of structural steels, which is known to increase the density of dislocations, has been shown to improve radiation tolerance^7^. However, just as often, materials with pre-existing defects have lower radiation tolerance. In contrast to metals, most inorganic nonmetallics, including Si^8^, Ge^9^, SiC^10^, and ZnO^11^, have shown reduced radiation tolerance with grain refinement, with GaN being a notable exception^12^.

Despite decades of research into the role of pre-existing defects in radiation damage formation, it is still not clear why some types of pre-existing disorder promote defect recombination while others inhibit it. Many possible mechanisms have been proposed. One possible explanation is that pre-existing defects such as surfaces, interfaces, or dislocations could preferentially annihilate either vacancies or interstitials, which can lead to an interstitial or vacancy rich conditions with an either enhanced or reduced rate of DA^13–15^. Molecular dynamics (MD) simulations in the past decade have clarified some of the atomistic processes involved. For example, MD results by Bai *et al*.^16^ suggest that grain boundaries exhibit a loading/unloading effect in which interstitial absorption and subsequent re-emission lead to highly efficient defect recovery. However, MD simulations are capturing only the initial stages of DA up to ~1 ns, while recent experiments have shown that, for many practical cases, the characteristic DA time scale (*τ*) is $\gtrsim 1$ *μ*s^17–24^.

Here, we report an experimental study demonstrating how pre-existing disorder influences defect interaction dynamics. We use a recently developed pulsed ion beam method^17–24^ to measure *τ* in Si with two representative types of pre-existing disorder: (i) point defect clusters and (ii) so-called “clamshell” defects consisting of a high density of dislocations. Our results demonstrate that *τ* and, hence, the rates of the dynamic defect recovery and point defect trapping, can be effectively controlled by such a defect engineering approach: *τ* increases in the presence of point defect clusters and decreases when clamshell defects are introduced into the lattice.

## Results and Discussion

**Figure 1:**
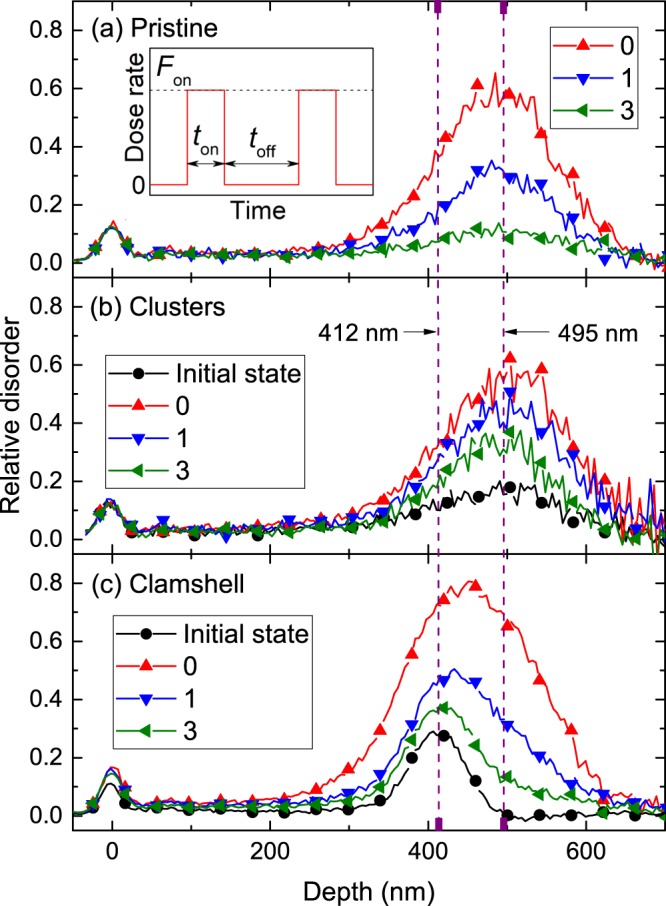
Selected depth profiles of relative disorder for pulsed 500 keV Ar ion beam irradiation of (**a**) pristine Si, (**b**) Si with pre-existing defect clusters created by continuous beam irradiation to a dose of 4.5 × 10^14^ cm^−2^, and (**c**) Si with the 500- °C-clamshell defect. Values of *t*_*off*_ (in milliseconds) are given in legends. The defect state prior to pulsed beam irradiation is shown by solid circles and labeled “Initial state” in (**b**) and (**c**). For clarity, only every 10th experimental point is depicted in all the profiles. The inset in (**a**) is a schematic of the time dependence of the instantaneous dose rate for pulsed beam irradiation, defining *t*_*on*_, *t*_*off*_, and *F*_*on*_. The total doses of pulsed Ar ions were 8.8×, 6.5×, and 8.0 × 10^14^ cm^−2^ in (**a**–**c**), respectively.

Figure 1 shows representative depth profiles of relative disorder for bombardment with continuous (*t*_*off*_ = 0 ms) and pulsed (*t*_*off*_ = 1 and 3 ms) ion beams for samples without any pre-existing disorder [Fig. 1(a), labeled “pristine”] and samples with defect clusters [Fig. 1(b)] and the 500- °C-clamshell defect [Fig. 1(c)]. The experimental conditions used to prepare such samples with pre-existing defects are summarized in Table 1 and described in the Methods section below. The inset in Fig. 1(a) shows a schematic of the time dependence of the dose rate in pulsed beam experiments and defines pulsing parameters *t*_*on*_, *t*_*off*_, and *F*_*on*_. For comparison, also shown by circles in Fig. 1(b,c) are damage profiles in samples with pre-existing cluster and clamshell defects prior to pulsed-beam bombardment. All the profiles in Fig. 1 are bimodal, with the first small peak at the sample surface and the second major peak in the crystal bulk. The bulk peak in Fig. 1(a,b) is at ~500 nm, corresponding to the maximum in the depth profile of vacancies ballistically generated by 500 keV Ar ion bombardment^25^. For the clamshell samples of Fig. 1(c), the bulk defect peak is at ~450 nm, which is closer to the position of the clamshell defect centered on ~410 nm.

**Table 1:** Summary of experimental conditions used to create samples with pre-existing lattice defects.

Pre-existing defect	Dose rate	Dose	Irradiation temp.	Annealing temp.	Annealing time
	(10^13^ cm^−2^ s^−1^)	(10^13^ cm^−2^)	(°C)	(°C)	(min)
Clusters	1.9	15–70	100	—	—
500- °C-clamshell	1.9	1.7	−196	500	45
600- °C-clamshell	1.9	1.7	−196	600	45

In all these cases, irradiation was done with continuous beams of 500 keV Ar ions.

**Figure 2:**
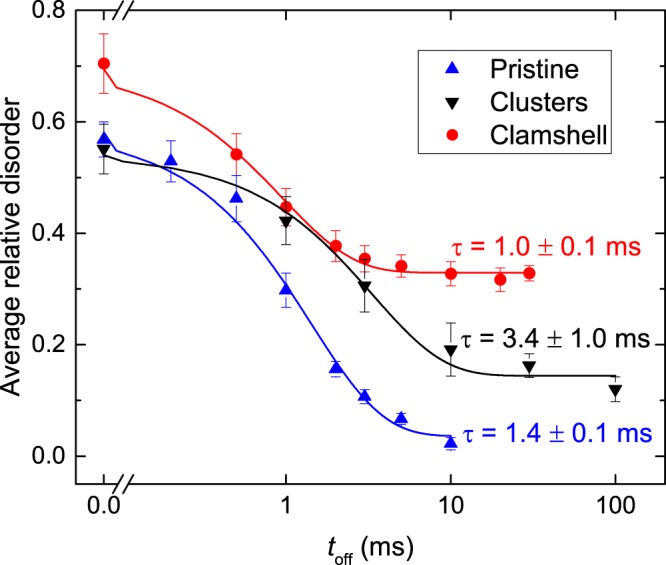
Average bulk relative disorder in Si bombarded at 100 °C with pulsed beams of 500 keV Ar ions as a function of the passive portion of the beam duty cycle (*t*_*off*_) for Si without pre-existing disorder (labeled “pristine”), Si with pre-existing defect clusters created by continuous beam irradiation to a dose of 4.5 × 10^14^ cm^−2^, and Si with the 500- °C-clamshell defect. Fitting curves with the first order decay equation are shown by solid lines.

Figure 1 further shows that the average relative bulk disorder (*n*, which is the height of the bulk damage peak) decreases with increasing *t*_*off*_ for all three sets of samples. This finding is better illustrated in Fig. 2, which summarizes *n*(*t*_*off*_) dependencies for these three sample sets. It is seen from Fig. 2 that, for all the cases, *n* monotonically decreases with increasing *t*_*off*_. Solid lines are fits of *n*(*t*_*off*_) dependencies via the Marquardt-Levenberg algorithm with the first order decay equation [*n*(*t*_*off*_) = *n*_∞_ + (*n*(0) − *n*_∞_)exp(−*t*_*off*_/*τ*)]. Here, *τ* is the characteristic decay time constant, and *n*_∞_ is relative disorder for *t*_*off*_ ≫ *τ*. For pristine Si and samples with clusters and the 500- °C-clamshell defect, *τ* was 1.4 ± 0.1, 3.4 ± 1.0, and 1.0 ± 0.1 ms, respectively.

The above results indicate that pre-existing lattice disorder strongly influences defect interaction dynamics. However, the *τ* measured by the pulsed beam method reflects DA processes occurring over a range of damage states, starting from the initial condition (in our case, either pristine Si or crystals with clusters or clamshell defects) to the final disorder level created by continuous beam bombardment with *t*_*off*_ = 0 ms. For example, when measuring *τ* in pristine Si (i.e., without any pre-existing disorder prior to pulsed beam bombardment) with a certain *n*(*t*_*off*_ = 0), we are essentially measuring *τ* averaged across the disorder level range from 0 to *n*(*t*_*off*_ = 0). The fact that defect relaxation dynamics is influenced by the presence of lattice defects (Fig. 2) suggests that *τ* should depend on the total dose in pulsed beam experiments.

**Figure 3:**
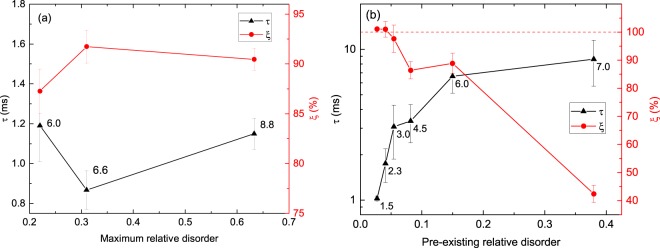
Dependence of (left axis) the defect relaxation time constant (*τ*) and (right axis) the dynamic annealing efficiency (*ξ*) on (**a**) the maximum average bulk relative disorder level (i.e., *n* for *t*_*off*_ = 0 ms) for Si without pre-existing defects and (**b**) on the level of average bulk relative disorder in Si with cluster defects present before pulsed ion beam irradiation. Numbers next to data points indicate, in (**a**), the total pulsed Ar ion doses used (in units of 10^14^ cm^−2^) and, in (**b**), ion doses (in units of 10^14^ cm^−2^) of continuous beam irradiation required to create such pre-existing disorder.

To test this hypothesis, we have measured the dependence of *τ* on the total ion dose for pulsed beam irradiation of pristine Si. Figure 3(a) (left axis) summarizes these results, plotting *τ* as a function of the maximum bulk relative disorder for *t*_*off*_ = 0 ms. It is seen that the resulting change in *τ* with increasing maximum bulk disorder (or the total irradiation dose) is negligible. This is in agreement with previous findings^19^ that *τ* is independent of the total dose for (pristine) Si irradiated at room temperature by 500 keV Ar ions. Hence, *τ* appears to be dominated by defect interaction processes that occur at relative disorder levels of $\lesssim 0.2$, characterized by a fast rate of defect relaxation, rather than by the much slower rate in a defective lattice for larger ion doses probed in the experiments summarized in Fig. 3(a).

To explore the role of pre-existing defect clusters on defect dynamics more systematically, Fig. 3(b) (left axis) plots *τ* as a function of the level of pre-existing disorder in Si with cluster defects, with irradiation doses used to create such pre-existing defect clusters shown next to data points (in units of 10^14^ cm^−2^). It is seen from Fig. 3(b) (left axis) that *τ* strongly and monotonically increases with increasing concentration of pre-existing clusters. Such an increase in *τ* (i.e., slowing of the defect relaxation dynamics) could be reflecting the trapping of migrating point defects at shallow energy wells of defect clusters, followed by subsequent de-trapping. Such trapping-detrapping processes, previously invoked to explain anomalous impurity diffusion in Si^26–28^, slow down defect relaxation and increase *τ* but do not necessarily involve defect recombination.

Effects of pre-existing disorder and the total dose used in pulsed beam experiments on defect recombination efficiency are better illustrated by measurements of the dynamic annealing efficiency (*ξ*). Figure 3(a) (right axis) shows *ξ* as a function of the maximum bulk relative disorder for *t*_*off*_ = 0 ms, while Fig. 3(b) (right axis) plots the dependence of *ξ* on the level of pre-existing disorder in Si with cluster defects. Here, we define *ξ* as (*n*(0) − *n*_∞_)/(*n*(0) − *n*_*pre*_), where *n*_*pre*_ is the level of pre-existing disorder in samples with cluster defects. It is seen from Fig. 3(a) (right axis) that the change in *ξ* with changing the total dose in pulsed beam experiments is negligible. In contrast, Fig. 3(b) (right axis) reveals a strong decrease in *ξ* from ~105 to ~40% with increasing relative level of pre-existing cluster defects from ~0.03 to ~0.38. Interestingly, for very small levels of pre-existing defect clusters of ~0.05, Fig. 3(b) (right axis) reveals *ξ* > 100%, which is due to ion-beam-induced annealing effects for ${t}_{off}\gg \tau$. As the level of pre-existing defect clusters increases above ~0.05, damage accumulation outweighs ion-beam-induced annealing effects, and *ξ* becomes <100%. The decrease in *ξ* with increasing concentration of pre-existing defect clusters reflects a decrease in the total fraction of defects participating in DA. These pre-existing defect clusters and/or amorphous zones provide sites for point defect trapping, resulting in either the formation of stable damage or the trap-mediated recombination of mobile point defects.

**Figure 4:**
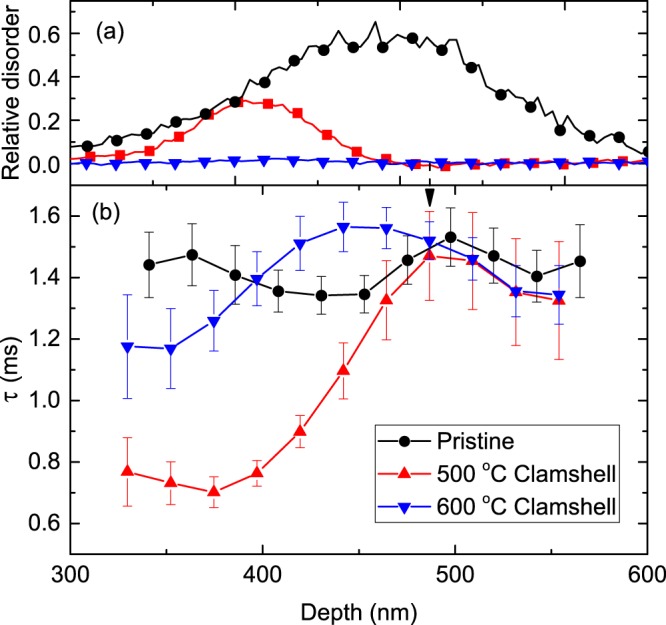
(**a**) Depth profiles of relative disorder in Si bombarded at 100 °C with a continuous beam of 500 keV Ar ions with a dose rate of $\sim 1.9\times {10}^{13}$ cm^−2^ s^−1^ to a dose of 8.8 × 10^14^ cm^−2^ (labeled “pristine”) and of Si with 500- and 600- °C-clamshell defects prior to pulsed beam irradiation. For clarity, only every 5th experimental point is depicted. (**b**) Depth dependence of the defect relaxation time constant (*τ*) for the three samples from (**a**) irradiated at 100 °C by a pulsed beam of Ar ions.

The above results (Figs 1–3) have revealed that defect relaxation processes are slowed down in the presence of point defect clusters and amorphous zones. Figure 2 also demonstrates a qualitatively opposite effect of *faster* defect relaxation in the presence of a different type of pre-existing disorder, the clamshell defect corresponding to a narrow buried region with a high density of dislocations formed where two crystallization fronts meet during thermally-induced recrystallization of a buried amorphous layer^14,29^. The role of clamshell defects is better illustrated in Fig. 4, showing depth profiles of disorder [Fig. 4(a)] and *τ* [Fig. 4(b)] in samples with and without 500- and 600- °C-clamshell defects. A comparison of Fig. 4(a,b) reveals that *τ* for pristine Si is approximately constant in the entire depth range probed, corresponding to the bulk damage peak. However, *τ* rapidly decreases in the depth range where the clamshell defects are present. As expected, *τ* for pristine Si and samples with clamshell defects are statistically indistinguishable at depth beyond ~480 nm, which is deeper than the position of both clamshells.

Figure 4(a) further shows that, compared to the 500- °C-clamshell defect, the 600- °C clamshell, prior to pulsed beam irradiation, is barely detectable by ion channeling. Despite a much lower concentration of dislocations, the 600- °C-clamshell defect shows a qualitatively similar effect on *τ* to that of the 500- °C clamshell [Fig. 4(b)]. The decrease in *τ* (and, hence, a corresponding increase in the DA rate) is due to the interaction of mobile defects with dislocations. Goldberg *et al*.^14^ have found that the clamshell defect is capable of preferentially nucleating damage and lowering the damage level of the surrounding lattice. The shift in the bulk damage peak, clearly visible in Fig. 1(c), is in agreement with such a preferential defect nucleation around the clamshell defect. However, the total irradiation dose to reach amorphization was similar in pristine Si targets and in samples with clamshell defects, suggesting that the details of the influence of pre-existing defects on damage buildup are non-trivially dependent on irradiation conditions. Such an invariance of the total dose to the presence of dislocations and a concurrent increase in the DA rate due to a more efficient point defect trapping at dislocations suggests that point defect trapping does not need to result in the growth of the dislocation band but could be related to faster defect recombination.

The above results illustrate an important point that *τ* reflects only the rate of defect relaxation dynamics, while *ξ* and the parameters describing the damage buildup dependence should be used to evaluate the partition between the ballistically-generated point defects that experience recombination or contribute to the formation of stable disorder measured after irradiation. We also point out that the experimental data alone, such as presented here, does not allow us to differentiate between contributions from different atomic-level DA processes. Future dedicated modeling and simulation studies, bench-marked against pulsed-beam data, are currently needed for a better understanding of the atomistics of DA in the Si lattice with pre-existing defect clusters.

## Summary

In summary, we have presented a method to experimentally study the dynamic interaction of mobile point defects with pre-existing lattice disorder. By using pulsed 500 keV Ar ion beams, we have studied the time constant of defect relaxation (*τ*) in Si at 100 °C with two representative types of pre-existing disorder: point defect clusters and clamshell defects. Results have revealed that *τ* increases with increasing concentration of pre-existing defect clusters. In contrast, dislocations associated with clamshell defects cause a significant reduction in *τ*. These changes to *τ* could be explained by a combination of the trapping and re-emission of mobile point defects at shallow energy wells associated with clusters, amorphous zones, and dislocations. These results demonstrate an experimental approach that could be used for designing and testing novel radiation tolerant materials. Our results could also be used to benchmark future modeling efforts aimed at identifying the atomistics of the dominant defect interaction processes.

## Methods

The 4 MV ion accelerator (National Electrostatics Corporation, model 4UH) at Lawrence Livermore National Laboratory was used for both ion irradiation and ion beam analysis. Float-zone grown (100) Si single crystals (with a resistivity of ~5 Ω cm) were bombarded with 500 keV^40^ Ar ^+^ ions at 7° off the [100] direction. As discussed elsewhere^23^, in these pulsed-beam experiments, the concentration of radiation-generated stable lattice defects largely exceeds the initial dopant concentration. In this case, the target is in a semi-insulating state, and point defects are expected to be in a neutral (rather than charged) state^23^. To improve thermal contact, the samples were attached to a Cu sample holder with conductive Ag paste. All irradiations described here, including experiments with continuous beams, were performed in a broad beam mode (rather than with rastered beams)^17^ with an instantaneous dose rate (*F*_*on*_) of ~1.9 × 10^13^ cm^−2^ s^−1^.

Table 1 summarizes the irradiation conditions used to prepare samples with three different types of pre-existing defects that we refer to as “clusters,” “500- °C-clamshell,” and “600- °C-clamshell.” The set of samples with pre-existing defects that we refer to as clusters was prepared by irradiation at 100 °C with a continuous 500 keV Ar ion beam to doses ranging from 1.5× to 7.0 × 10^14^ cm^−2^. Although the primary disorder process under these conditions is the accumulation of point defect clusters, we note that the formation of amorphous zones also occurs, particularly for larger doses in the regime of overlapping cascades when stable damage levels, measured by ion channeling, are $\gtrsim 10$ %^30,31^.

Samples with clamshell defects were created according to a procedure described by Goldberg *et al*.^14^ by first irradiating at −196 °C with a continuous 500 keV Ar ion beam to a dose of 1.7 × 10^13^ cm^−2^. This created a buried amorphous layer. Next, these samples were annealed at 500 or 600 °C for 45 minutes in an Ar atmosphere, causing recrystallization of the buried amorphous layer from the two amorphous/crystalline interfaces. The clamshell defect, consisting of a dense network of dislocations, forms in the region where the two crystallization fronts meet. Such clamshell defects were the subject of previous systematic electron microscopy studies^14,29^. We refer to these two sets of samples as 500- °C- and 600- °C-clamshells.

Radiation defect dynamics in samples with and without such pre-existing defects was measured at 100 °C by the pulsed beam method when the total Ar ion dose was split into a train of equal square pulses with a pulse duration (*t*_*on*_) of 1 ms. Adjacent pulses were separated by *t*_*off*_, which was varied between 0.1 and 100 ms. A more detailed description of the experimental arrangement can be found elsewhere^17–19^. A discussion of effects of *F*_*on*_ on pulsed beam measurements was given in ref.^19^.

The dependence of lattice damage on *t*_*off*_ was studied *ex-situ* at room temperature by ion channeling with 2 MeV^4^ He^+^ ions incident along the [100] direction and backscattered into a detector at 164° relative to the incident beam direction. Raw channeling spectra were analyzed with one of the conventional algorithms^32^ for extracting depth profiles of relative disorder. Values of average bulk relative disorder (*n*) were obtained by averaging depth profiles of relative disorder over 10 channels (~30 nm) centered on the bulk defect peak. Error bars of *n* are standard deviations. A detailed discussion of the measurement of depth profiles of radiation damage by ion channeling can be found in refs^32,33^. The depth profile of ballistically-generated vacancies was calculated with the TRIM code (version SRIM-2013.00, full cascade calculations)^25^ with an atomic concentration of 5 × 10^22^ at. cm^−3^ and a threshold energy for atomic displacement of 15 eV^34–36^.
